# Impact of adjustment for differential testing by age and sex on apparent epidemiology of SARS-CoV- 2 infection in Ontario, Canada

**DOI:** 10.1186/s12879-025-10968-6

**Published:** 2025-04-23

**Authors:** Savana Bosco, Amy Peng, Ashleigh R. Tuite, Alison Simmons, David N. Fisman

**Affiliations:** 1 Dalla Lana School of Public Health, University of Toronto, Room 686, 155 College Street, ON M5 T 3M7 Toronto, Canada; 2 Centre for Immunization Programs, Public Health Agency of Canada, Room 686, 155 College Street, ON M5 T 3M7 Toronto, Canada

**Keywords:** SARS-CoV-2, Epidemiology, Pandemics, Testing, Statistical methods, Validation

## Abstract

Communicable disease surveillance typically relies on case counts for estimates of risk, and counts can be strongly influenced by testing rates. In the Canadian province of Ontario, testing rates varied markedly by age, sex, geography and time over the course of the SARS-CoV-2 pandemic. We applied a standardization-based approach to test-adjustment to better understand pandemic dynamics from 2020 to 2022, and to better understand when test-adjustment is necessary for accurate estimation of risk. Case counts were adjusted for under-testing using a previously published standardization-based approach that estimates case numbers that would have been expected if the entire population was tested at the same rate as most-tested age and sex groups. After adjustment for under-testing, estimated case counts increased threefold and test-adjusted cases correlated better with SARS-CoV-2-attributed death than crude reported cases. Test-adjusted epidemic curves suggested, in contrast to reported case counts, that the first two pandemic waves were equivalent in size, and identified three distinct pandemic waves in 2022, due to the emergence of Omicron variants. Under-reporting was greatest in younger individuals, with variation explained partly by testing rates and prevalence of multigenerational households; test-adjustment resulted in little change in the epidemic curve during time periods when per capita testing rates exceeded 5.5%. We conclude that standardization-based adjustment for differential testing by age and sex results in a different understanding of the epidemiology of SARS-CoV-2 in Ontario. This methodology may offer a means of deriving adjusted estimates of infection incidence from surveillance data, accounting for fluctuations due to changing test practices.

**Supplementary Information:**

The online version contains supplementary material available at 10.1186/s12879-025-10968-6.

## Introduction

The 2019 emergence of SARS-CoV- 2 resulted in a global pandemic with severe impacts on mortality, life expectancy, population health status, and economies [1–9]. While the acute phase of the pandemic appears to have subsided, this highly virulent airborne pathogen remains endemic worldwide. The true impact of the pandemic on population health continues to be debated, as does the likely impact of the virus’ ongoing endemic circulation. Understanding the pathogen’s impact depends on a clear vision of disease epidemiology, which in turn depends on accurate analysis of public health surveillance data.

Early in the pandemic we observed that the estimated incidence of SARS-CoV- 2 infection in Ontario, Canada, was strongly predicted by frequency of PCR testing; in other words, groups such as older adults, who were tested more intensively, appeared to have higher SARS-CoV- 2 incidence than groups tested less frequently (such as young males and children of both sexes) [10]. We developed a simple, regression-based approach that permitted adjustment for testing frequency, such that it became possible to estimate the incidence of infection that *would have* been observed in a given age/sex group had it been tested at the same rates as the most highly tested population group. We found that adjusting for test frequency resulted in a very different view of the pandemic; one in which younger individuals (and in particular, males aged 20–29) represented a far larger share of infections than was observable in unadjusted data [10].

Our earlier analysis was restricted to the period from initial SARS-CoV- 2 emergence in Ontario (which we dated to March 2020, when community transmission was first recognized) to December 2020 [10]. However, this early period preceded the widespread emergence of novel viral variants of concern (VOC) and use of vaccination to control the pandemic. Indeed, Mitchell et al. have divided Canada’s SARS-CoV- 2 into six distinct periods based on dominant circulating VOC and disease incidence, an approach which also partially captures the timing of SARS-CoV- 2 vaccine roll-out in Canada [11].

Our objectives were: to extend our earlier analysis, to evaluate the differences between epidemic curves generated using unadjusted and test-adjusted case counts, both for the pandemic overall and for distinct, individual waves that occurred between March 2020 and September 2022; and to generate age- and sex-specific epidemic curves, to quantify the degree to which cases are likely to have been under-recognized due to under-testing in different age and sex groups. We also sought to identify testing thresholds above which test adjustment made little difference to perceived epidemic activity.

### Data sources

We evaluated disease incidence using population-based SARS-CoV- 2 infection data from the Ontario Case and Contact Management System (CCM), a data system used by Ontario’s 34 public health units for public health management of notifiable diseases [10]. The case definition for SARS-CoV- 2 during the period under evaluation required a positive nucleic acid amplification test assay (e.g., real time PCR) from an accredited laboratory [12]. Positivity on rapid antigen-based tests were not included in the provincial SARS-CoV- 2 case definition. CCM included data on age (10-year intervals) and sex of case patients and date a positive SARS-CoV- 2 PCR was reported; as this last data element was complete, we used it as a surrogate for case date [10]. Laboratory testing volumes were obtained from the Ontario Laboratories Information System (OLIS), which includes testing and reporting dates for all PCR tests performed in the province, including tests performed in the public health laboratory system, hospital system and private laboratories. As such OLIS is believed to be a complete record of SARS-CoV- 2 PCR testing in Ontario during the period under study. We counted the first test record when a person had multiple tests on a given day; however, subsequent testing of that person could be incorporated into test counts [10]. SARS-CoV- 2 attributable deaths were defined as cases in which a fatal case outcome was recorded.

### Classification of waves

We classified the SARS-CoV- 2 pandemic in Ontario by slightly modifying the approach of Mitchell et al., who identified six distinct waves based on disease activity and dominant circulating viral variant of concern: waves 1 and 2 (wild-type dominant, with wave 1 ending on August 31, and wave 2 from September 1 2020 to February 28, 2021; wave 3 (mixed Alpha/Beta/Gamma variants, March 1 to June 30, 2021); wave 4 (Delta variant dominant, from July 1 to December 25, 2021); and Omicron dominant waves 5 and 6 (from December 26 2021 to March 19, 2022, and after March 20, 2022, respectively) [11]. For each of these waves, we evaluated overall per capita testing by age and sex, and identified the most tested group during each wave.

### Adjustment for under-testing

We used meta-regression-based methods to adjust case counts in other age and sex groups for under-testing, estimating the case rates that would have been expected if these groups were tested at the same rate the most tested group. This method is described in detail elsewhere [10], but briefly requires that a standardized case ratio (SCR), and standardized testing ratio (STR), be estimated weekly, by public health unit, for each age- and sex-group, with incidence and testing rates in the most tested group used in the denominator of these ratios. We used meta-regression to model the relationship between standardized case and testing rates across strata defined by age, sex, and health unit. This approach allowed us to incorporate variance estimates as weights, giving more influence to strata with greater precision and accounting for heteroskedasticity in the data.

To account for temporal changes in testing practices, we performed a wave-specific test adjustment. For each pandemic wave, we identified the age–sex group with the highest testing rate (“most-tested” group) and used its testing rate as the reference to standardize observed case counts. Specifically, for each wave, we calculated a standardized case ratio (SCR) for each age–sex stratum by comparing its observed case incidence to the incidence that would be expected if that stratum were tested at the same rate as the most-tested group in that wave. For 5 of six waves, the most-tested group was females aged 80 and over. However, during the fourth wave, the most tested group was male children under 10 years of age, and this group was used as the referent for that period. Although this approach results in different scaling across waves, it reflects the reality that testing volumes and target groups varied substantially over time. The wave-specific adjusted case estimates were then concatenated to generate an overall epidemic curve. This piecewise adjustment ensures that under-testing is corrected relative to the contemporaneous testing landscape, avoiding distortions that would arise from applying a single global reference. To evaluate the impact of this analytic choice we also performed a sensitivity analysis in which females aged 80 and over were used as the referent group for the entire pandemic period. We evaluated the impact of switching referent groups by comparing test-adjusted case estimates for wave 4 using the two different approaches and found that the adjusted case estimates generated using the two approaches were similar (Supplementary Appendix).

Using wave-specific SCR and STR, we constructed age- and sex-specific meta-regression models stratified by public health unit. These models used log-transformed values of SCR and STR, such that:

$$\text{E}\left[1\text{n}\left(\text{SCR}\_\text{i}\right)\right]=\upalpha_i+\upbeta_i *1\text{n}\left(\text{STR}\_\text{i}\right)$$

As ln(STR_i_) is zero when a given age and sex group is tested at the same rate as the most tested group, the model intercept α_i_ can be interpreted as the natural log of the standardized case ratio that would be expected in the presence of equal testing. This SCR can then be multiplied by observed infection incidence in the most tested group, to generate an estimate of test-adjusted incidence. While there are a number of modeling approaches that could be used to create such estimates, we opted to use meta-regression models due to ease of incorporating inverse-variance weights for SCR and STR estimates when generating adjusted case counts. Weekly test-adjusted case estimates by age and sex for each public health unit were generated in this way. Overall test-adjusted epidemic curves were created by summing adjusted case numbers. We generated 95% confidence intervals for test-adjusted case counts by applying the predicted standardized case ratios—derived from the intercepts of our meta-regression models—and their standard errors to the reported case counts for each age–sex–public health unit–week stratum. To estimate uncertainty in the total number of adjusted cases across strata, we summed the adjusted case estimates and used bootstrapping (1000 replications) to generate 95% confidence intervals for the weekly total.

### Analysis

We compared crude and test-adjusted epidemic curves for Ontario graphically. To assess the relationship between these curves and SARS-CoV- 2-attributable mortality, we normalized the curves by dividing by their standard deviations and evaluated their association with deaths normalized in the same way. Given that the median and mean lags from test positivity to death were approximately 2 weeks, we calculated Spearman correlation coefficients using a two-week lag (Supplementary Appendix). We also compared the fit of linear regression models predicting deaths using either crude or adjusted case counts as independent variables. To formally compare correlation coefficients, Fisher's Z-transformation was applied [13], with statistical significance set at *p* < 0.05. Bootstrap methods (1,000 replications) were used to generate 95% confidence intervals for correlation coefficients. Linear regression models were compared using R^2^ and Akaike Information Criteria (AIC). As immunization might have attenuated the association between adjusted cases and deaths, we also calculated Spearman correlation coefficients for each pandemic wave individually (Supplementary Appendix).

While Spearman correlation and linear regression provided initial insights, these approaches are limited in capturing the temporal complexity and cumulative effects of exposure over time. Neither method accounts for delayed, overlapping, or distributed impacts of case counts on mortality (Supplementary Appendix). To overcome these limitations, we employed distributed lag nonlinear models (DLNMs), which model associations between an exposure (case counts) and an outcome (deaths) across a range of lags (1 to 8 weeks) [14–16]. DLNMs are particularly suited for epidemiological data where delayed effects are expected. They use a cross-basis function to define the relationship along both the exposure and lag dimensions [14–16]. For the exposure–response relationship, we used cubic smoothers to allow for flexible, non-linear associations, accommodating potential diminishing or accelerating impacts of case counts on deaths. Smooth functions were also used for the lag structure to capture the gradual onset and dissipation of risk over time. We compared DLNMs using adjusted and crude case counts based on R^2^, AIC, and Vuong’s test for statistical comparison [17].

We evaluated the ratio of reported cases to test-adjusted case estimates by age group, sex, public health unit, and time period. We denoted this ratio a “adjustment ratio” (denoted AjR to avoid confusion with “attack rate”). As overall AjR were < 1 (i.e., test-adjusted cases exceeded reported cases we calculated the standard error of ln(AjR) as the square root of ((1/reported cases) + (1/(test-adjusted cases—reported cases))).

We quantified the relative magnitude of AjR by age, sex, period and public health unit, and evaluated statistical significance for differences between groups, using negative binomial regression models, using reported cases as the dependent variable, and adjusted case estimates as model offsets. Models included linear, quadratic and cubic time trend terms, as well as fast Fourier transforms (FFT) to capture disease seasonality. Age groups were treated as indicator variables, with the oldest age group (80 +) used as the referent. Public health units were also included as indicator variables, with the public health unit with the median AjR (Kingston) used as the referent. Interaction between age and sex was evaluated using multiplicative interaction terms. Such a model produces “incidence rate ratios” which can be interpreted as a *relative* AjR (RAjR) by age, sex, period, public health unit, and so on.

Heterogeneity in AjR across public health units was evaluated using meta-analytic methods, with meta-regression used to quantify the extent to which such heterogeneity could be explained by variation in test rates, health resources, vaccine uptake, urbanicity, and socioeconomic and demographic characteristics of public health units. We used non-long-term care hospital beds per capita for each public health unit as a surrogate for local health resources; these were obtained from the Canadian Institute for Health Information [18]. Vaccination coverage was defined as total cumulative SARS-CoV- 2 vaccine doses per capita over the period under study; vaccination data were obtained from COVAXON as described elsewhere [19, 20]. Urbanicity effect was evaluated by comparing effects within the “Greater Toronto/Hamilton Area” (GTHA), the province’s largest population concentration, to effects outside the GTHA. Socioeconomic and demographic characteristics of each public health unit were obtained from Statistics Canada’s 2021 Census data [21]; these included mean population age; proportion of population over age 64; proportion of multigenerational households; income inequality (Gini coefficient based on after tax household income); percent visible minorities; percent of residents identifying as Indigenous; percent of residents who are new immigrants; percent of residents who own a home; unemployment rate; proportion of residents with low income; median after tax household income; proportion of residents with educational attainment less than high school graduation; and proportion of residents who are Canadian citizens. The associations between each of these public health unit-level characteristics and AjR were evaluated with univariable meta-regression models; factors with P ≤ 0.20 were evaluated in a multivariable meta-regression model built using backwards elimination.

We noted that although adjusted case counts were, overall, higher than crude case counts, test adjustment resulted in little change in the overall epidemic curve during periods when testing rates were high, and in some age- and sex- groups, and time periods adjusted case counts were, in fact, lower than crude reported case counts. We evaluated the relationship between this phenomenon and group-specific testing rates using logistic regression models. Analyses were performed in Stata version 15.1, R Studio version 2024.12.1, and QGIS version 3.32.3-Lima, using the final dataset made available to researchers by the Ontario Provincial Government (dataset created September 4, 2022). Aggregate case and test counts, population denominators, and public health unit characteristics needed to perform meta-regression, can be obtained at [22]. Further guidance on performance of analysis can be obtained by contacting Dr. Fisman. The study was approved by the Research Ethics Board of the University of Toronto.

## Results

**Fig. 1:**
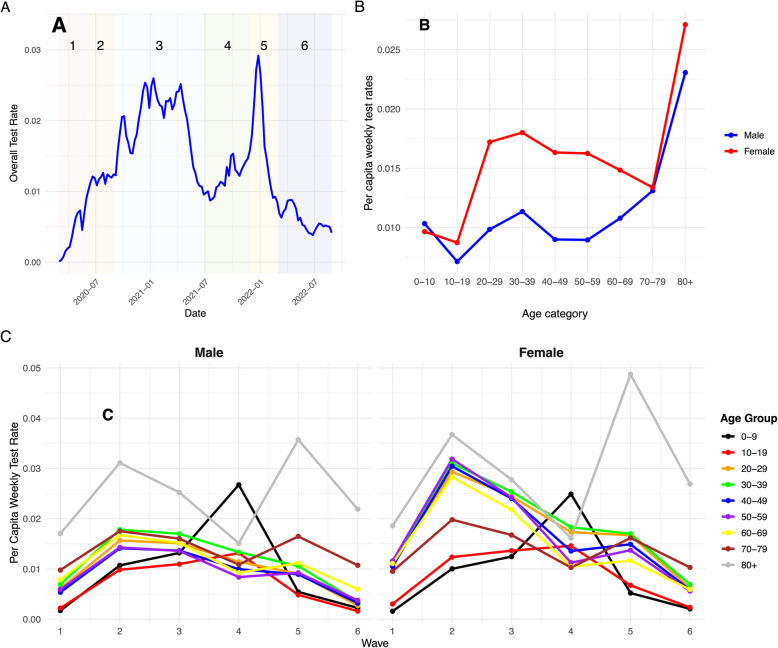
Weekly Rates of Testing for SARS-CoV- 2 in Ontario, Canada, March 2020 to August 2022. **A** Overall weekly per capita test rates by date of test report. Shading and numbers denote distinct pandemic waves as defined by Mitchell et al. [11]. **B** Average per capita weekly test rates by age group for males and females over the entire study period. **C** Average per capita weekly test rates for males (left panel) and females (right panel) by pandemic wave. Colors of curves correspond to individual age groupings; for all waves except wave 4 the most tested group is females aged 80 and over; in wave 4 the most tested group was 0- to 9-year-old males

Between March 1, 2020 and September 4, 2022, 22.82 million PCR tests were performed for SARS-CoV- 2 in the province of Ontario with 1,377,146 (6.03%) of these tests positive. Testing rates varied over time, with two distinct peaks: from December 2020 to March 2021, when per capita test rates were above 2% per week; and in December 2021 and January 2022, when per capita testing rates approached 3% per week (Fig. 1). Females aged 80 and over were the most-tested group over the study period, and were tested at a rate of 2.7% per week; males aged 10–19 were tested at the lowest rates (0.7% per week) (Fig. 1). When we evaluated testing by age and sex for each pandemic wave, we found that for 5 of 6 identified waves, females aged 80 and over were the most tested group; however, for wave 4 (Delta-variant dominant) the most tested group was males aged < 10 years (Fig. 1).

**Fig. 2:**
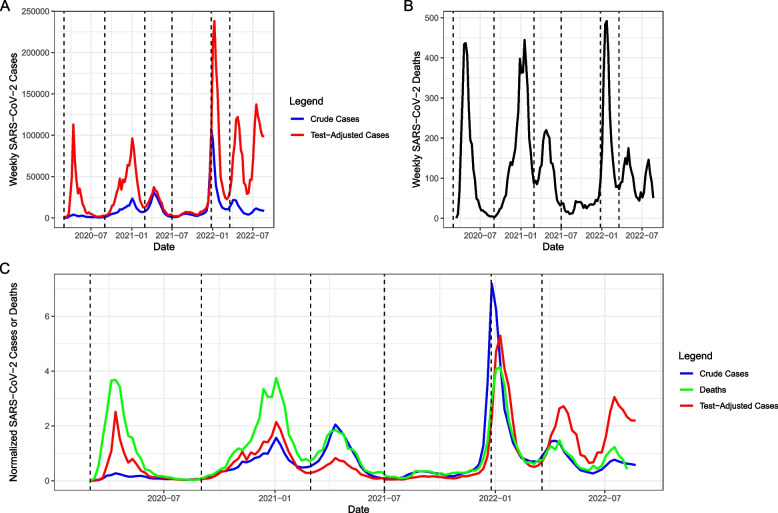
Reported and Test-Adjusted Epidemic Curves, and Correspondence with SARS-CoV- 2 mortality in Ontario, Canada. **A** Crude reported (blue) and test-adjusted (red) case counts over the course of the SARS-CoV- 2 pandemic. Early and late pandemic waves are far larger with test adjustment, reflecting low overall rates of testing. **B** SARS-CoV- 2 deaths in Ontario over time. Similar numbers of deaths in the first and second waves, and three distinct waves of deaths with emergence of Omicron variants in the later phase of the pandemic, correspond with patterns seen in the test-adjusted epidemic curve, but not with patterns seen in the reported case epidemic curve. **C** Crude reported (blue) and test-adjusted (red) case curves, and weekly deaths (green) backdated by 14 days to account for lags between case reporting and death, are normalized by dividing by their standard deviations (SD), and are presented on a single graph. Vertical dashed lines denote boundaries between pandemic waves

Cumulative case counts after adjustment were 3.1 times higher than crude reported cases (4.3 million cases vs. 1.43 million cases). After adjustment for under-testing based on most-tested group in each wave, we were able to construct a test-adjusted epidemic curve and compare it graphically to the reported epidemic curve. Test adjustment resulted in a different appearance to the epidemic curve at the beginning and end of the pandemic, when testing per capita was at its lowest (Fig. 2). In particular, the first wave of the pandemic in spring 2020, and the second wave that autumn, appeared equivalent in magnitude after test adjustment. Test adjustment also markedly increased the apparent magnitude of the first wave caused by Omicron variant emergence and identified two subsequent large waves of infection caused by Omicron in spring and summer of 2022. By contrast, test-adjusted and unadjusted curves, for the mid-pandemic period when testing was most intense, were almost identical.

Graphical comparison of the test adjusted epidemic curve to a plot of lagged SARS-CoV- 2 attributed deaths by week demonstrated similar magnitude of the 1 st and 2nd pandemic waves for both test-adjusted cases and deaths. Three distinct waves of both adjusted cases and deaths were seen in the 5 th and 6 th pandemic waves, after emergence of Omicron variants (Fig. 2).

Spearman rank correlations between lagged normalized deaths and adjusted cases was higher for adjusted cases (*p* = 0.82, 95% CI 0.73 to 0.90) than for crude cases (*p* = 0.76, 95% CI 0.64 to 0.87), but these differences were not statistically significant (*P* = 0.19). Wave-by-wave correlations are presented in the Supplementary Appendix. Linear regression models predicting lagged normalized deaths showed higher R^2^ and better model fit for adjusted cases (R^2^ = 0.43, model AIC = 299) than for crude cases (R^2^ = 0.36, model AIC = 313).

**Fig. 3:**
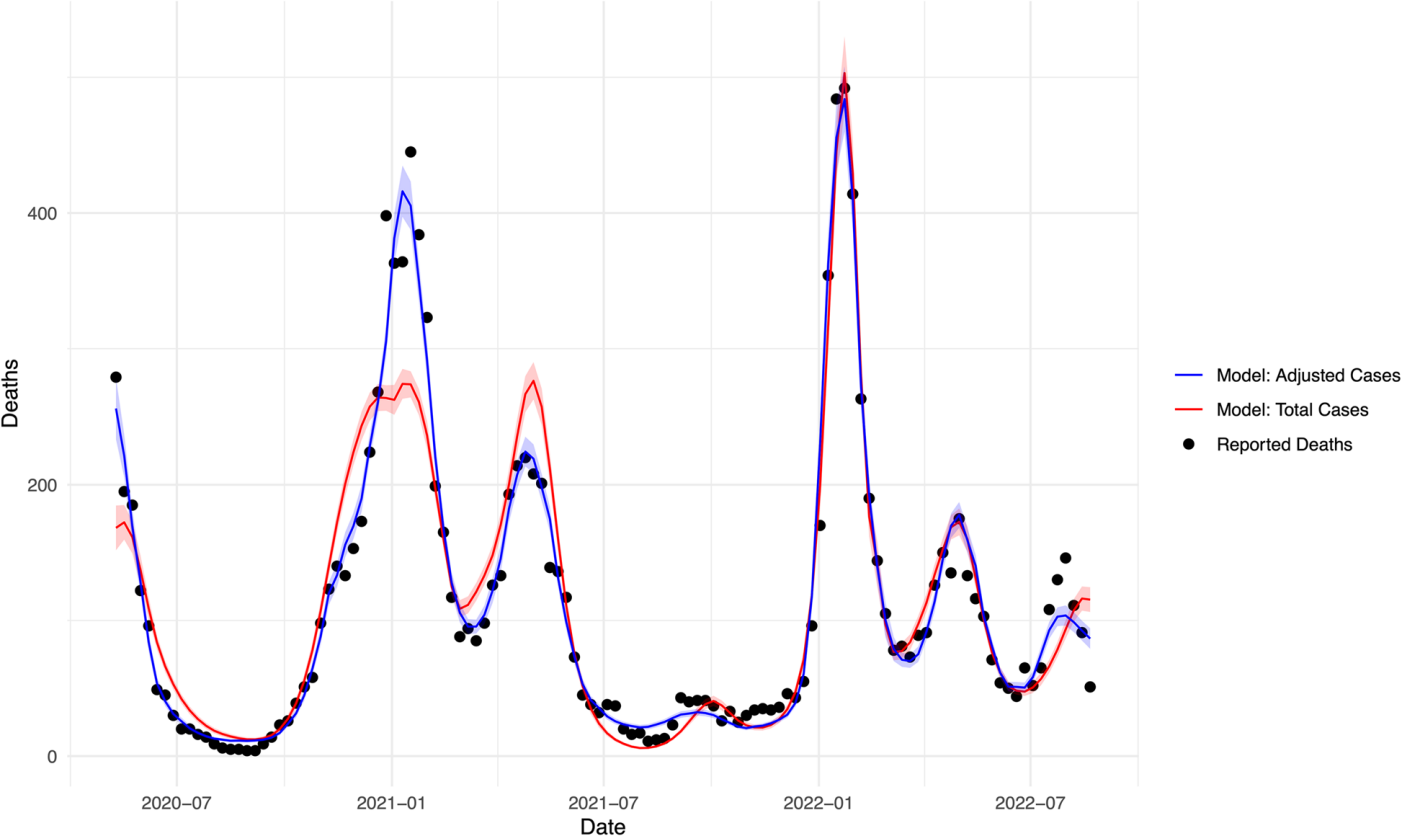
Comparison of Predicted and Actual COVID- 19 Deaths Over Time in Ontario, Canada. The figure displays actual reported deaths (black dots) alongside predicted deaths from distributed lag nonlinear models (DLNMs) using total reported cases (red curve) and test-adjusted cases (blue curve) as predictors. Shaded areas represent the 95% confidence intervals for each model. The adjusted case model demonstrated superior fit, with stronger alignment to observed deaths, particularly during periods of limited testing

In distributed lag nonlinear models (DLNMs), test-adjusted case counts provided a better fit for predicting deaths compared to reported case counts. The DLNM using adjusted cases demonstrated a higher McFadden's R^2^ (R^2^ = 0.912) and lower AIC (AIC = 1051) than the model using reported cases (R^2^ = 0.849, AIC = 1792). Differences in model fit were statistically significant (*P* < 0.001 by Vuong’s test). Graphical comparisons also showed that the model based on adjusted cases more closely aligned with observed mortality trends over time (Fig. 3).

When we calculated crude adjustment ratios (AjR), we found that AjR were highest in females aged 20–59 but below 1 in all other age and sex groups, and AjR approached 1 during the third and fourth pandemic waves (Supplementary Appendix). Negative binomial models identified significant (*P* < 0.001) interaction between age group and sex in relative under-reporting, with relative AjR (RAjR) higher (i.e., closer to 1) in females than in males. Patterns of relative reporting, adjusted for age, sex, period and geography, were similar in negative binomial models to patterns identified via calculation of crude AjR (Table 1).

**Table 1:** Relative adjustment ratios for SARS-CoV- 2 by age, sex and time period

Covariate	Relative Adjustment Ratio	95% Confidence Intervals	*P*-value
**Age, Male**			
0 to 9	0.487	0.462–0.514	< 0.001
10 to 19	0.560	0.532–0.590	< 0.001
20 to 29	0.982	0.933–1.032	0.471
30 to 39	1.052	1.000–1.106	0.051
40 to 49	0.958	0.910–1.008	0.101
50 to 59	0.936	0.889–0.984	0.01
60 to 69	0.973	0.925–1.024	0.304
70 to 79	0.872	0.828–0.920	< 0.001
80 and over	1.117	1.060–1.177	< 0.001
**Age, Female**			
0 to 9	0.541	0.513–0.570	< 0.001
10 to 19	0.720	0.683–0.758	< 0.001
20 to 29	1.661	1.581–1.745	< 0.001
30 to 39	1.646	1.567–1.730	< 0.001
40 to 49	1.473	1.401–1.547	< 0.001
50 to 59	1.258	1.197–1.323	< 0.001
60 to 69	1.077	1.023–1.134	0.004
70 to 79	0.744	0.706–0.784	< 0.001
80 and over (referent)	1.000	–-	–-
Time Period (Pandemic Wave)			
1 (referent)	1	–-	–-
2	0.569	0.533–0.607	< 0.001
3	1.139	1.045–1.241	0.003
4	0.878	0.789–0.977	0.017
5	0.529	0.468–0.597	< 0.001
6	0.390	0.339–0.448	< 0.001XX

Estimates are derived from a negative binomial regression model, with reported case counts used as dependent variable and test-adjusted counts used as model offsets. Models are also adjusted for public health unit and time trends

Male and female estimates are presented separately due to significant interaction between age and sex

Overall, AjR across public health units ranged from 0.11 (in Timiskaming) to 0.69 (in Peel); the overall pooled AjR was 0.31, with significant heterogeneity (*P* < 0.001) (Supplementary Appendix). In univariable meta-regression models, testing rate, fraction of multigenerational households, percent of population identifying as Indigenous, and non-long term care hospital beds per capita were each associated with URR with P ≤ 0.20. Only testing rate and multi-generational households remained in our final multivariable meta-regression model due to our use of backwards elimination. We found that each percentage increase in the proportion of the population tested resulted in a 2.55-fold increase in the AjR, while each percentage increase in multigenerational households resulted in a 1.08-fold increase in AjR (Table 2 and Supplementary Appendix).

**Table 2:** Univariable and multivariable meta-regression models of variation in adjustment ratios (AjR) by public health unit

Univariable models				Multivariable model		
**Variable**	**AjR**	**95% CI**	*P*-value	**AjR**	**95% CI**	*P*-value
Weekly testing rate	1.93	0.99–3.77	0.054	2.55	1.31–4.94	0.007
Hospital beds per capita	0.98	0.96–1.01	0.128	–-	–-	–-
Cumulative vaccination	0.98	0.76–1.26	0.872	–-	–-	–-
Mean age	0.98	0.93–1.03	0.418	–-	–-	–-
Proportion of population over 64	0.99	0.96–1.02	0.411	–-	–-	–-
Household size	1.33	0.78–2.27	0.289	–-	–-	–-
Proportion of multigenerational households	1.05	0.98–1.12	0.164	1.08	1.01–1.16	0.019
Gini coefficient	0.22	0.00–67.93	0.598	–-	–-	–-
Proportion visible minority	1.00	0.99–1.01	0.607	–-	–-	–-
Proportion identifying as Indigenous	1.01	1.00–1.03	0.166	–-	–-	–-
Proportion immigrants	1.00	0.99–1.01	0.572	–-	–-	–-
Proportion homeowners	1.00	0.98–1.02	0.816	–-	–-	–-
Unemployment rate	1.04	0.98–1.10	0.219	–-	–-	–-
Prevalence of low income	0.99	0.93–1.04	0.577	–-	–-	–-
Log of median household income	1.17	0.30–4.65	0.813	–-	–-	–-
Proportion of residents with less than high school education	1.00	0.97–1.04	0.804	–-	–-	–-
Proportion Canadian citizens	0.99	0.96–1.02	0.503	–-	–-	–-

NOTE: *AjR* Adjustment ratio

Predictors with *P*-values less than or equal to 0.20 in univariable meta-regression models were included as candidate predictors in a final multivariable model, which was reduced using backwards elimination; only weekly testing rate and proportion of multigenerational households were retained in the final model

**Fig. 4:**
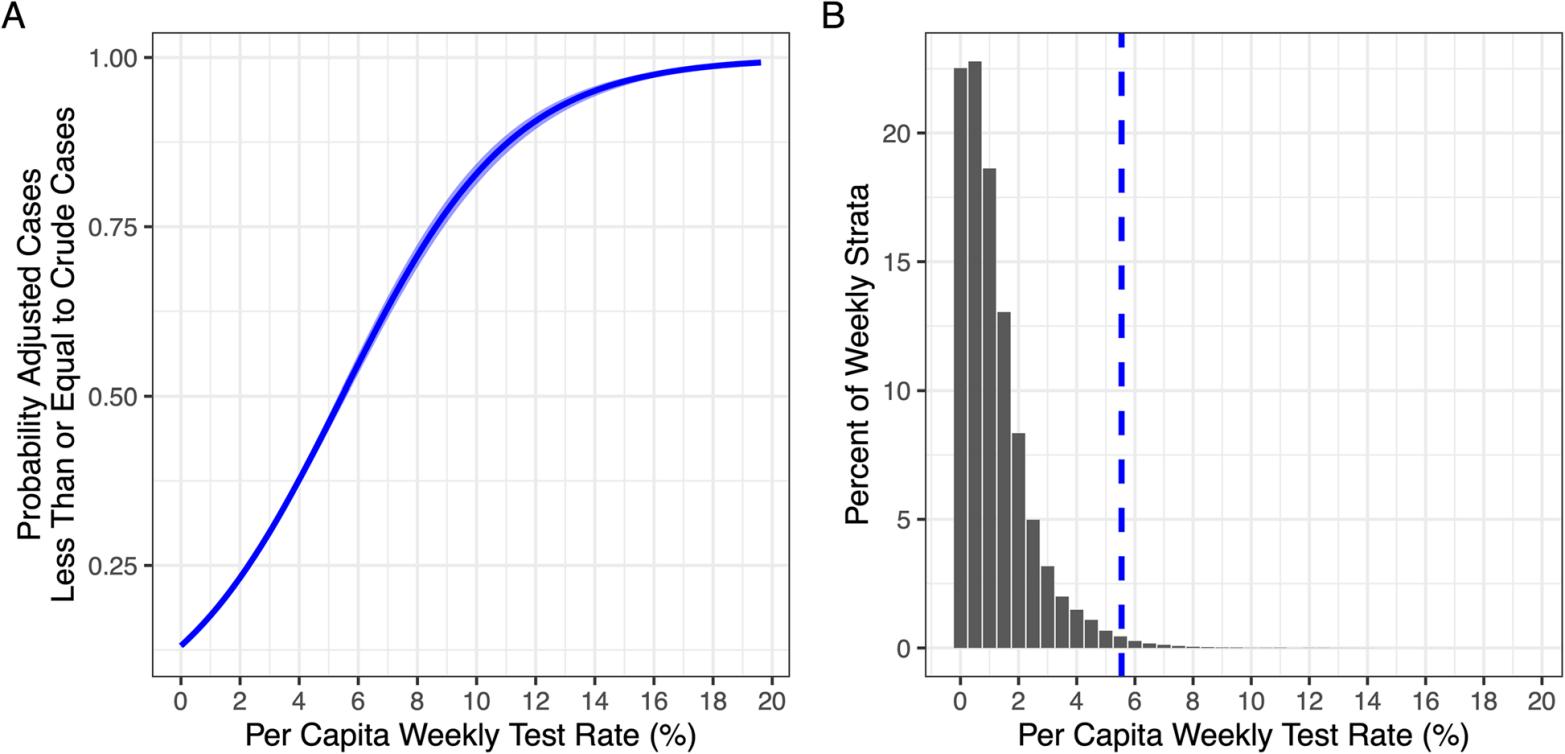
Testing Frequency and Likelihood of Test-Adjusted Case Counts Less Than or Equal To Reported Case Counts. **A** Predictions from a logit regression model of probability that adjustment ratio (ratio of reported cases to adjusted cases is greater than or equal to 1) with increasing per capita testing rates. Solid blue curve represents probabilities and shaded area represents 95% confidence intervals. Probability of an adjustment ratio greater than or equal to 1 exceeds 50% when per capita weekly testing rates exceed 5.5%. **B** Histogram demonstrates the relative frequency of per capita test rates by week, public health unit, biological sex and age. For most strata, test rates are below the 5.5% threshold, denoted by the vertical dashed blue line

We evaluated the relationship between adjusted case counts equal to, or lower than, crude reported case counts and testing rates using logistic regression. As shown in Fig. 4, for every one percent increase in per capita weekly testing, the odds of adjusted case counts being less than or equal to crude case counts increased by 39% (95% CI 37% to 41%, *P* < 0.001). The weekly per capita test rate at which adjusted case counts were equally likely to be higher or lower than reported case counts was 6.26% (95% CI 6.08% to 6.47%).

## Discussion

While case counts are the mainstay of public health surveillance systems, it is often forgotten that case counts are heavily influenced by testing volume. The tendency to focus testing on individuals and groups at highest risk for severe outcomes during the SARS-CoV- 2 pandemic led us to develop a standardization-based approach to test adjustment, which captures log-linear relationships between testing rates and case rates, by indexing them to incidence in highly tested age and sex groups [10]. Our test-adjustment approach accounts for differences in testing intensity across subgroups, which can mask underlying differences in infection risk. While all individuals were initially susceptible to SARS-CoV- 2 infection, exposure patterns varied across age groups due to differences in behaviors and non-pharmaceutical interventions (NPIs) [10, 23].

While initial exploratory analyses comparing predictive validity for mortality evaluating crude correlations between reported case rates and adjusted case rates and reported deaths found a non-significant trend towards improved correlation with adjusted deaths using a fixed two-week lag, we were able to validate this approach against reported deaths using DLNM that used a more realistic lag structure. Although both reported cases and adjusted cases were predictive of lagged death, both the significantly better fit of the adjusted case model, and graphical evaluation of model predictions, demonstrated the predictive validity of this approach for better approximation of true population-level burden of infection (reported and unreported).

In applying this method across the duration of the SARS-CoV- 2 pandemic in Ontario, we find that test-adjustment results in a changed picture of risk during the pandemic, particularly during early and late pandemic periods when test volumes were low. Notably, we found that the apparent differences in the magnitude of the first two pandemic waves likely reflected changing testing volumes rather than changing disease epidemiology, consistent with the similarity in mortality associated with each of these waves. Test adjustment also made it clear that Ontario experienced three distinct waves of illness due to Omicron variants (BA1, BA4, and BA5) from winter to summer of 2022. The latter two waves were obscured by falling testing rates from January 2022 onwards, but the test-adjusted epidemic curve is consistent with three distinct waves of SARS-CoV- 2 death present in Ontario’s data.

We found that testing rates resulted in substantial heterogeneity in the extent to which cases were under-reported by age-group and sex. Overall, cases in females were more likely to be identified than cases in males, not only because of intensive testing in the oldest females who represented the majority population in the province’s long-term care facilities. We also found that there was less under-reporting in younger adult females (ages 20 to 59), which may reflect different health behaviors and knowledge [23], but may also reflect the demographics of Ontario’s female-majority healthcare workforce. By contrast, adjustment ratios were notably low in children and younger male adults over the study period, despite higher rates of testing in schools in pandemic wave 4, suggesting that infection was under-recognized in these groups.

We also found considerable heterogeneity in under-reporting by health units; this heterogeneity was partially explained by variation in testing rates across health units, but increased prevalence of multi-generational households in a health unit was also associated with a significant narrowing of the gap between reported and test-adjusted case counts. This finding has considerable face validity: if public health practice throughout the pandemic was to advise testing of household contacts of SARS-CoV- 2 cases, occurrence of cases in multi-generational households would, by definition, have resulted in enhanced testing across age groups.

While adjusting for under-testing provided a different perspective on the pandemic compared to reported case counts, our analysis also identified population testing frequency thresholds beyond which such adjustment was unnecessary. During periods of high testing—particularly in pandemic waves 3 and 4—adjusted and reported case curves were nearly identical, suggesting that crude case counts can reasonably represent disease dynamics when testing rates are sufficient. Notably, during high-testing periods, crude case counts sometimes exceeded test-adjusted estimates, likely due to random variation at higher thresholds. Additionally, serological studies in Ontario [24] indicate that young adults experienced substantially higher attack rates than older individuals, suggesting that, in these groups, crude case counts may have closely approximated true incidence. In such instances, test-adjusted estimates—which assume uniform testing at the highest observed rate—could slightly underestimate true incidence.

Our approach advances traditional test-adjustment methods in public health surveillance. Many systems, including Canada’s FluWatch, rely on test positivity rates to account for variations in testing volume [25]. However, test positivity has limitations as an index of risk: testing volume and positivity interact bidirectionally (i.e., rising risk increases testing, and increased testing identifies more cases with varying risk profiles). Our method directly estimates case numbers as if all groups were tested at the rate of the most-tested group, avoiding the need for additional assumptions about the size of the tested population.

Although sero-epidemiological methods are often considered the gold standard for estimating infection burden, they have important limitations in the Ontario SARS-CoV- 2 context. These include: (i) limited sensitivity/specificity of individual assays [26]; (ii) reliance on potentially unrepresentative populations such as blood donors [26]; (iii) seroreversion [26, 27]; (iv) difficulty distinguishing infection- from vaccine-induced antibodies [26, 28]; and (v) reduced interpretability in the context of widespread, repeated infections.

The UK’s Office for National Statistics COVID- 19 Infection Survey provided a gold-standard approach for direct infection incidence estimation via regular household testing [29]. However, such studies are resource-intensive, and few jurisdictions have the infrastructure, capacity, or political will to implement them. Our method offers a scalable alternative for settings where serosurveys and large-scale longitudinal testing are unavailable, enabling more accurate retrospective assessments of epidemic dynamics and informing public health policy.

As with any observational research, our study has limitations. Our approach does not directly estimate infection incidence but instead estimates what observed case incidence would have been if all population groups had been tested at the same intensity as the most-tested group. While we have partially validated this method by assessing the alignment of test-adjusted case waves with mortality waves during periods of underreporting, external validation using wastewater and serological data is necessary and ongoing. Future work aimed at estimating true incidence will likely require integrated approaches incorporating serological, case, and mortality data. Additionally, we cannot assess the generalizability of our approach beyond Ontario or Canada, and our analysis is limited to the period before September 2022, when relevant data became unavailable [30].

In summary, we applied a standardization-based approach to population-based case data from the SARS-CoV- 2 pandemic in Ontario, Canada, and found that test-adjustment resulted in a different view of the pandemic during periods when testing rates were low. In particular, test adjustment was helpful in understanding the distribution of deaths over time by demonstrating that low case counts early and late in the pandemic represented under-testing rather than low incidence of infection. Further refinement of this approach by incorporation of testing data into public health surveillance may be possible through application to other disease processes [31] and in other jurisdictions.

## Supplementary Information

Supplementary Material 1.

## Data Availability

Aggregate case and test counts, population denominators, and public health unit characteristics needed to perform meta-regression, can be obtained at https://figshare.com/articles/dataset/Data_and_code_needed_to_recreate_Impact_of_Adjustment_for_Differential_Testing_by_Age_and_Sex_on_Apparent_Epidemiology_of_SARS-CoV-2_Infection_in_Ontario_Canada_/24243181. Further guidance on performance of analysis can be obtained by contacting Dr. Fisman.

## Abbreviations

AjR: Adjustment Ratio
AIC: Akaike Information Criterion
CI: Confidence Interval
COVID- 19: Coronavirus Disease 2019
DLNM: Distributed Lag Nonlinear Model
PCR: Polymerase Chain Reaction
PHU: Public Health Unit
SARS-CoV- 2: Severe Acute Respiratory Syndrome Coronavirus 2
SCR: Standardized Case Ratio
STR: Standardized Test Ratio
Vuong’s test: Statistical test for model comparison
