# Supplementary material for: Impact of adjustment for differential testing by age and sex on apparent epidemiology of SARS-CoV- 2 infection in Ontario, Canada
Source: BMC Infect Dis. 2025 Apr 23;25:589. doi: 10.1186/s12879-025-10968-6 (PMC12020243; doi:10.1186/s12879-025-10968-6)
Supplement: Supplementary file 1 — Supplementary Material 1. [file 12879_2025_10968_MOESM1_ESM.docx]

**Supplementary Appendix**

1. **Sensitivity Analysis on Different Referent Groups**

For most of the pandemic period, women aged 80 and over were the most tested group. However, in wave 4, male children under 10 were the most tested group. We evaluated the effects of using women as the most tested group throughout the pandemic as compared to our method of concatenating the epidemic curve, varying the referent group such that 5 of 6 waves used women aged 80 and over, and wave 4 used male children under age 10. The results are presented below.

It can be seen that when males aged < 10 were used as the referent group, adjusted cases (blue curve) were lower than reported cases (maroon curve) for most of the pandemic, as children were relatively under-tested outside of wave 4. Using highly-tested women aged 80 and over resulted, as would be expected, in substantially higher adjusted case rates. During the fourth pandemic wave, when all age groups were highly tested, there was little difference between reported cases, adjusted cases using males aged < 10 as the referent, and adjusted cases using females 80 and over as the referent, as this was a period of high rates of testing. Nonetheless, as shown in the graph below, using the most tested group (males under 10) as the referent for wave resulted in the highest adjusted case count during this wave, and an adjusted case count that exceeded reported cases.

1. **Lags from Case Identification to Death**

In order to identify appropriate lags for analyses evaluating the relationship between case counts and subsequent deaths, we used Ontario’s Case Contact Management (CCM) dataset, as described elsewhere (1-5). The graph below plots lag between SARS-CoV-2 test positivity and death date for 14,021 individuals with documented death date. The analysis was truncated at May 25, 2022 due to possible delays in death reporting. The graph below is restricted to death dates occurring up to 100 days after test positivity. Median time to death was 11 days (red dashed line); mean was 16 days (brown dashed line); the 25^th^ and 75^th^ percentiles were 5 and 18 days respectively (dashed blue lines). The distribution shows marked right-skewing.

1. **Spearman Correlation Coefficients by Pandemic Wave**

In addition to estimating overall Spearman correlation coefficients for reported cases and lagged deaths, and adjusted cases and lagged deaths, we evaluated coefficients wave by wave. The table below presents correlation coefficients with bootstrapped 95% confidence intervals, P-values for non-independence of cases and deaths, and P-values comparing Spearman correlations for adjusted and reported cases, estimated using Fisher's Z-transformation. On a wave-by-wave basis, correlations estimated with reported cases, and adjusted cases, were similar for all waves except wave 6.

| **Wave** | **Reported Cases** | | | | **Adjusted cases** | | | | **P-value (based on Fisher Z-transformation)** |
| --- | --- | --- | --- | --- | --- | --- | --- | --- | --- |
|  | **Spearman's rho** | **LCL** | **UCL** | **P for non-independence** | **Spearman's rho** | **LCL** | **UCL** | **P for non-independence** |  |
| Overall | 0.758 | 0.644 | 0.872 | <0.001 | 0.820 | 0.735 | 0.905 | <0.001 | 0.188 |
| 1 | 0.877 | 0.689 | 0.970 | <0.001 | 0.889 | 0.729 | 0.974 | <0.001 | 0.847 |
| 2 | 0.971 | 0.884 | 0.995 | <0.001 | 0.971 | 0.899 | 0.994 | <0.001 | 1.000 |
| 3 | 0.983 | 0.920 | 1.000 | <0.001 | 0.977 | 0.896 | 1.000 | <0.001 | 0.674 |
| 4 | 0.910 | 0.810 | 1.000 | <0.001 | 0.883 | 0.720 | 0.955 | <0.001 | 0.647 |
| 5 | 0.923 | 0.646 | 1.000 | <0.001 | 0.972 | 0.799 | 1.000 | <0.001 | 0.271 |
| 6 | 0.853 | 0.677 | 0.935 | <0.001 | 0.554 | 0.233 | 0.876 | 0.001 | 0.048 |
| **NOTE:** UCL, LCL, upper confidence limit and lower confidence limit, estimated with bootstrapping (N = 1000 replications). | | | | | | | | | |

Correlation between reported cases and deaths may reflect the fact that when testing rates were lower, individuals at higher risk of mortality (e.g., older individuals) were prioritized for testing. In wave 6, reported cases remained highly correlated with lagged deaths (rho 0.85), but correlation with adjusted cases was weaker (rho 0.55), with the difference between estimates statistically significant (P = 0.048). The weaker correlation between adjusted cases and deaths in the final pandemic wave may reflect protective effects of widespread vaccination.

1. **Additional Outputs from Distributed Lag Non-Linear Models**

We employed Distributed Lag Non-Linear Models (DLNMs) to characterize the delayed association between both reported and test-adjusted case counts and weekly death counts over 1 to 8-week lag periods. Main model results are presented in the Results section. We also evaluated lag-response plots associated with elevated case counts. The lag-response plots below relative risk (RR) of death across lag periods for case counts set at the 75th percentile. For adjusted cases, the RR increases most markedly for cases at lags of 1-4 weeks. For reported cases effects are seen at lags of 1-6 weeks. Protective effects (at 5-6 weeks for adjusted cases and 7-8 weeks for reported cases) may represent harvesting.

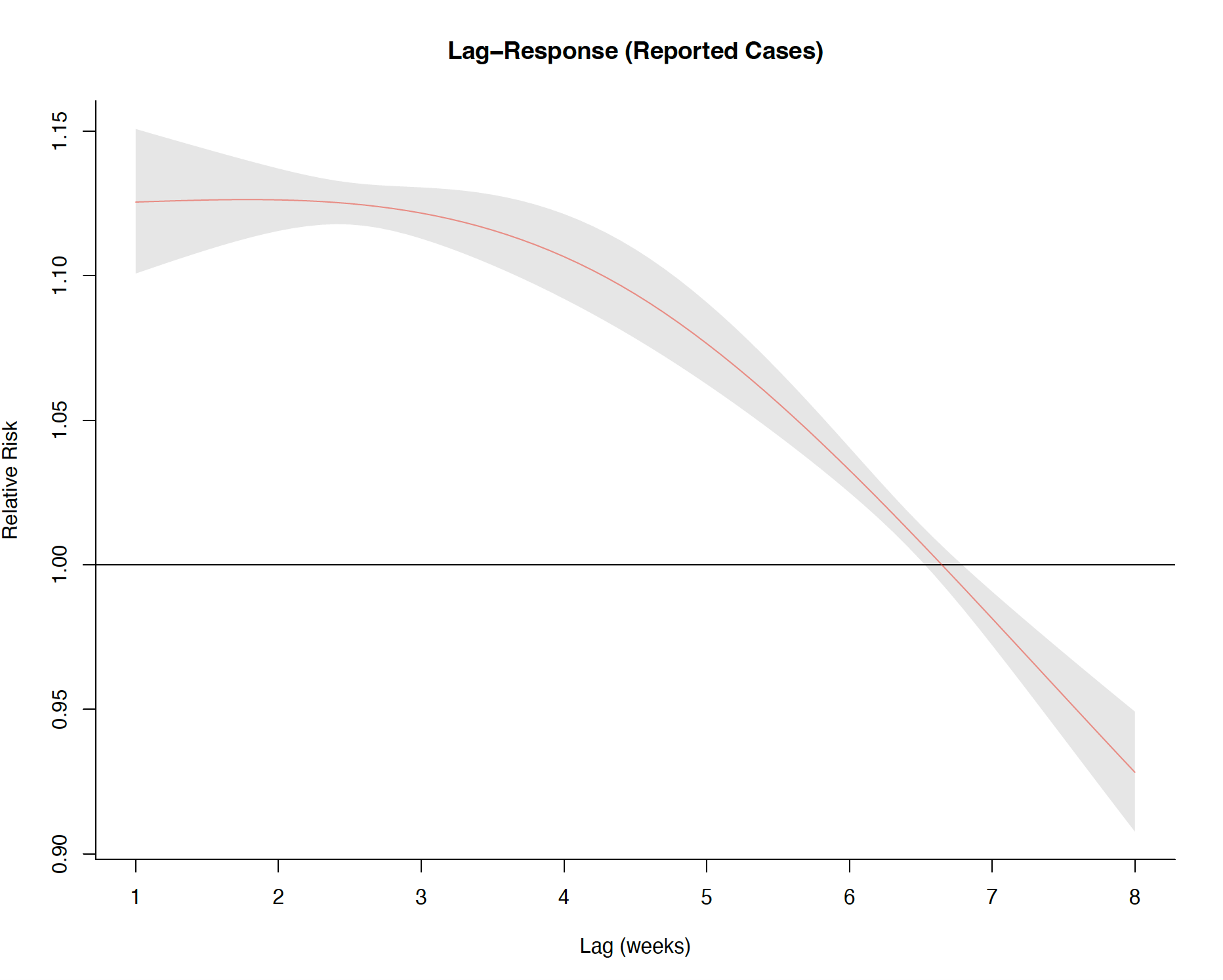


Exposure-response plots display cumulative RR of death across the range of case counts, aggregated over all lag periods (1-8 weeks). For both adjusted and reported cases, a dose-response relationship between increasing case counts and risk of death is seen.


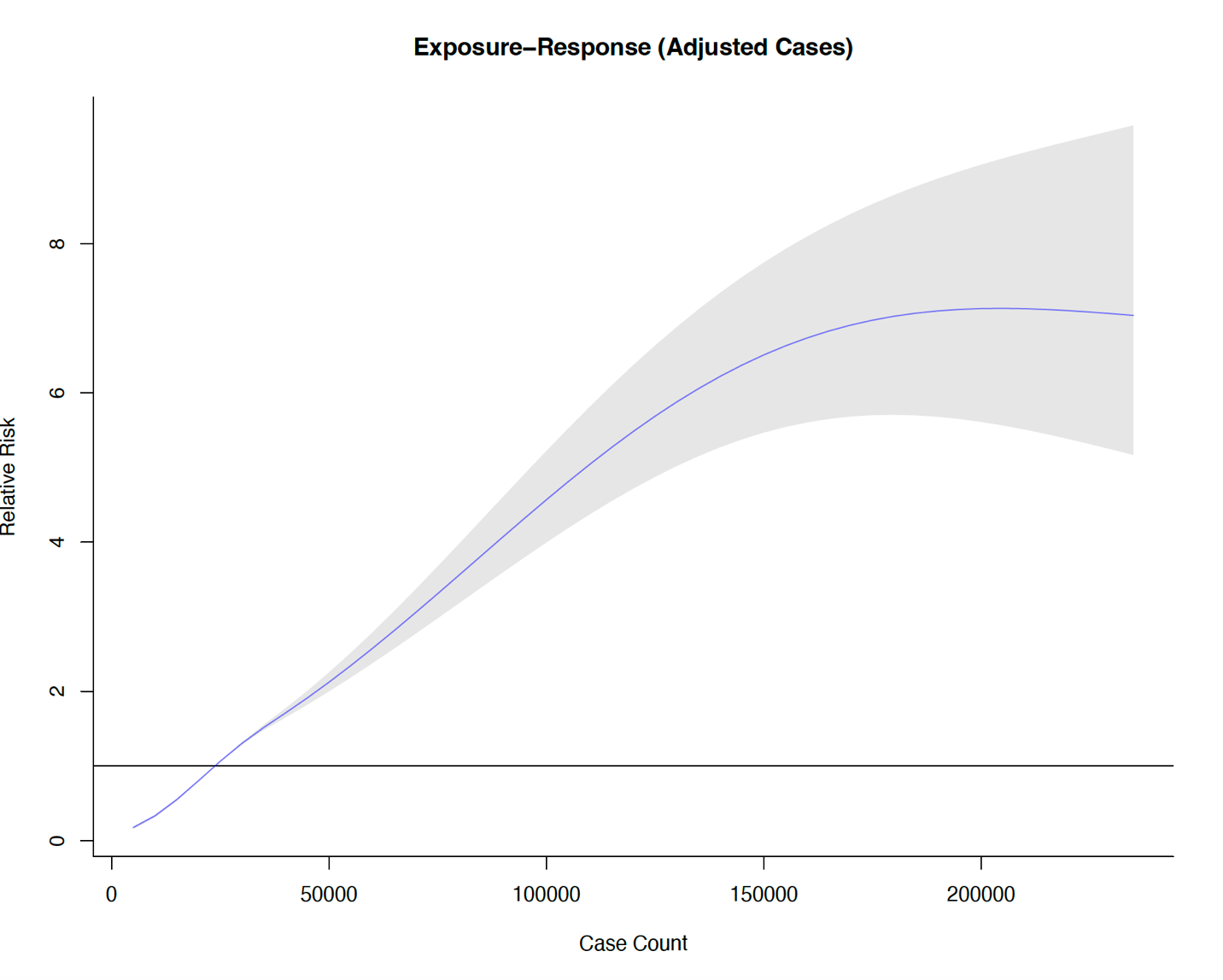


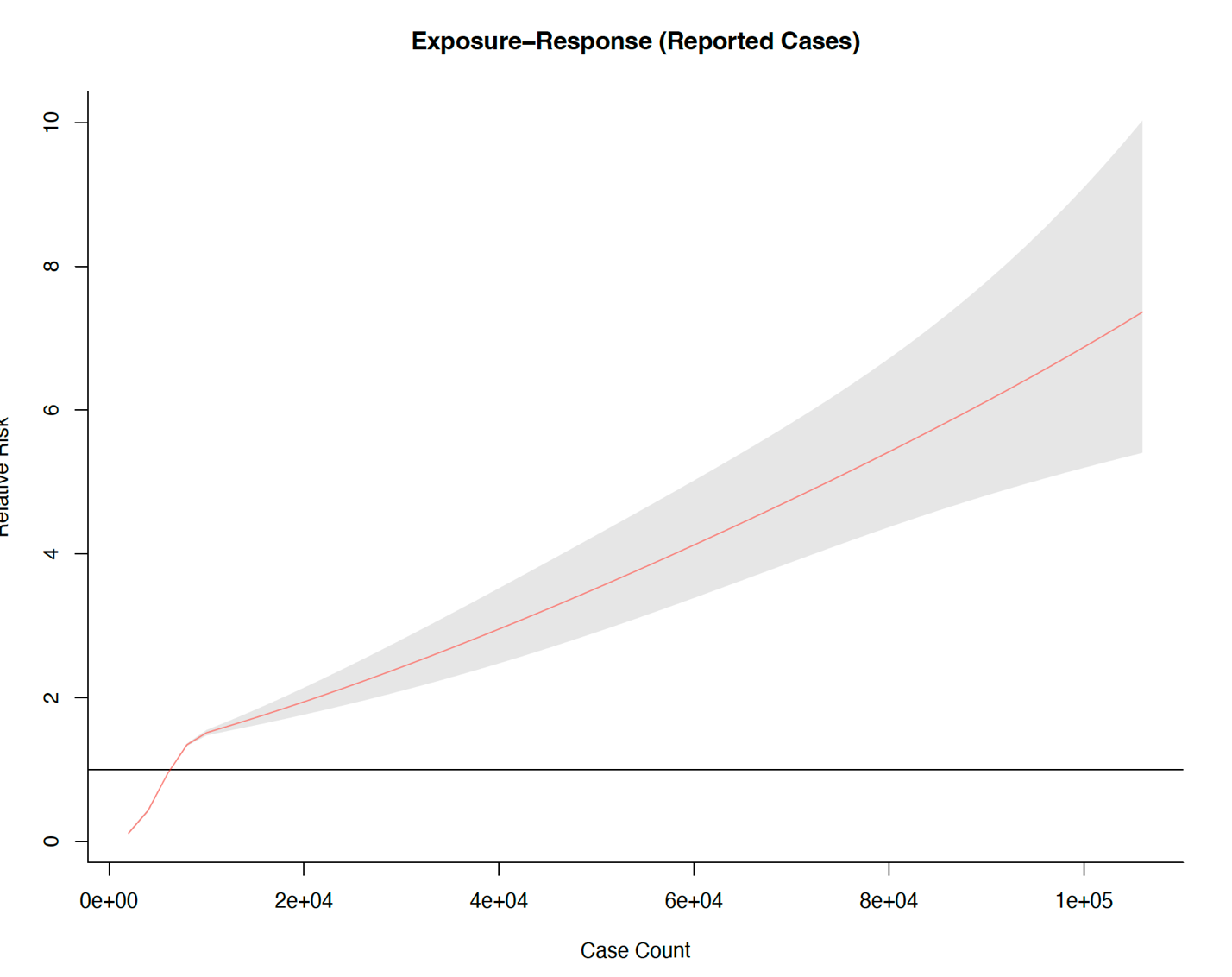


We plotted 3-dimensional surfaces that depict relative risks (Z-axis) across a range of lags (1-8 weeks) and case counts. For both adjusted cases and reported cases relative risk increased with shorter lags and higher case counts.


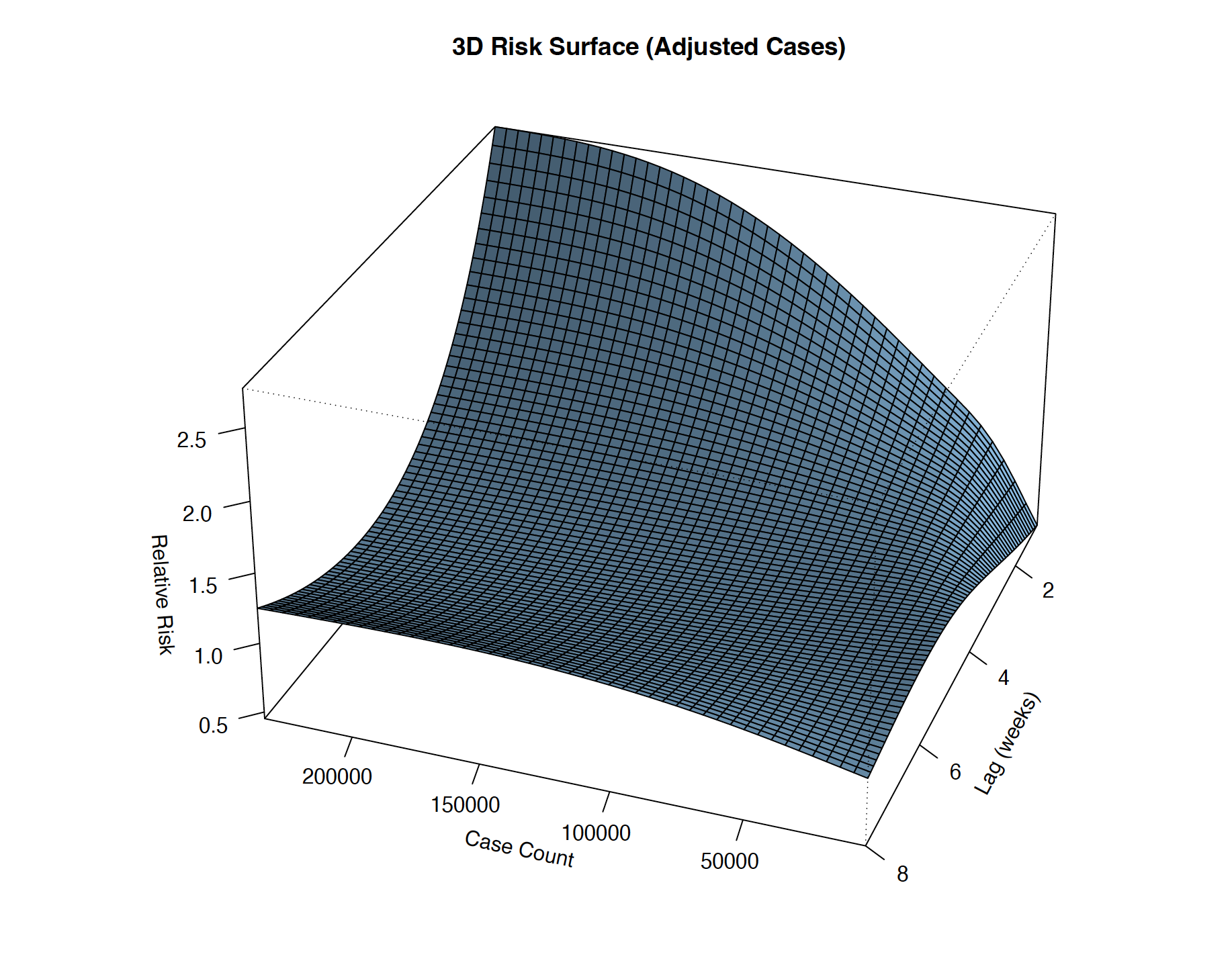


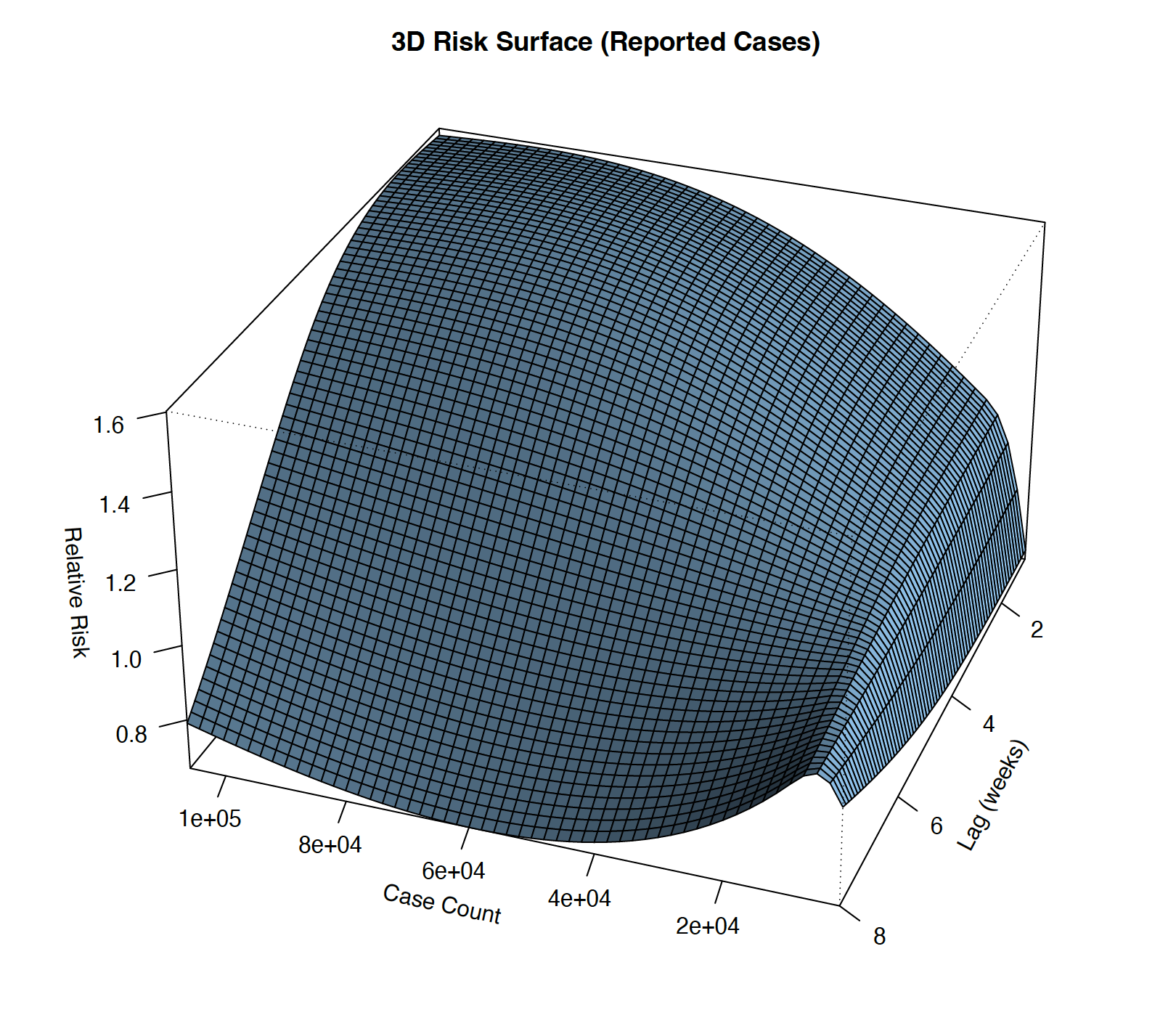


In summary, DNLM demonstrated the ability of both adjusted case counts and reported case counts to predict mortality across a range of lags. As noted in our Results section, the use of adjusted cases improved model fit and predictive ability.

**5. Adjustment Ratios and Average Weekly Testing Rates in Ontario Public Health Units**

(A) Average weekly SARS-CoV-2 testing rates (%) by health unit in Southern Ontario. (entire province is presented in inset). Darker regions had higher rates of testing. (B) Adjustment ratios by health unit in Southern Ontario (entire province in inset). Higher (darker) regions have higher adjustment ratios, meaning that unadjusted case counts are relatively closer to test-adjusted case counts than in other health units. Lighter regions have lower adjustment ratios, indicating a larger relative gap between reported case counts and test-adjusted case counts.

**6. Adjustment Ratios by Age, Sex and Time Period**

Panel A (left) shows adjustment ratios (Y-axis), defined as the ratio of reported cases to test-adjusted cases, over the study period by age-grouping (X-axis) and sex. Red bars represent females; blue bars represent males. Whiskers represent confidence bounds. Higher adjustment ratios indicate a smaller relative gap between reported cases and test-adjusted cases. Panel B (right) shows adjustment ratios for the population as a whole in each of six distinct pandemic waves. During waves 3 and 4, in the presence of high rates of testing, adjustment ratios were close to 1. Note that these adjustment ratios differ from

**7. Forest Plot of Adjustment Ratios by Ontario Health Unit**

Health unit names are listed on the left size of the figure; adjustment ratios (“effect size”, or “ES”) are listed on the right with 95% confidence intervals. Box sizes are inversely proportional to variance of estimates. Ratios range from 0.11 in Timiskaming to 0.69 in Peel, with a mean adjustment ratio of 0.31 across all health units, and substantial between health unit heterogeneity.

**8. Observed and Predicted Adjustment Ratios and Per Capita Testing**

Circles represent observed (red) and predicted (blue) adjustment ratios (AjR) for SARS-CoV-2 by Ontario public health unit. Predicted AjR are derived from a meta-regression model, which also adjusts for prevalence of multi-generational households by public health unit. Size of circles is inversely proportional to the variance of the estimate. Straight lines are lines of best fit for observed (red) and predicted (blue) estimates.
